# Characteristics, treatment, and outcome of recurrent gastro-oesophageal adenocarcinoma after perioperative chemotherapy and radical resection

**DOI:** 10.2340/1651-226X.2026.44264

**Published:** 2026-01-11

**Authors:** Anders Christian Larsen, Susy Shim, Lene Bæksgaard, Per Pfeiffer, Marianne Nordsmark, Rasmus Brøndum, Jan Reiter Sørensen, Anne Krejbjerg Motavaf, Morten Ladekarl

**Affiliations:** a Department of Oncology, Clinical Cancer Research Center, Aalborg University Hospital, Aalborg, Denmark; b Department of Gastrointestinal Surgery and Department of Clinical Medicine, Aalborg University Hospital, Aalborg, Denmark; c Department of Clinical Medicine, Aalborg University, Aalborg, Denmark; d Department of Oncology, Rigshospitalet, Copenhagen, Denmark; e Department of Oncology, Odense University Hospital, Odense, Denmark; f University of Southern Denmark, Odense, Denmark; g Department of Oncology, Aarhus University Hospital, Aarhus, Denmark; h Department of Clinical Medicine, Faculty of Health, Aarhus University, Aarhus, Denmark; i Center for Clinical Data Science (CLINDA), Aalborg University and Aalborg University Hospital, Aalborg, Denmark

**Keywords:** Cancer recurrence, gastro-oesophageal adenocarcinoma, national cohort study, perioperative chemotherapy, survival analysis

## Abstract

**Background:**

Evidence of treatment of patients with relapse following multimodal treatment for oesophageal, gastro-oesophageal junctional and gastric adenocarcinoma is almost absent.

**Methods:**

In a nationwide consecutive cohort of 202 patients, radically resected after perioperative chemotherapy (CTx) and followed-up without scheduled imaging, we identified 89 patients with recurrence within 12 years. We registered prior clinico-pathological and treatment characteristics, alarming symptoms, work-up, recurrence patterns, treatment of recurrence, and outcome.

**Results:**

Median time to recurrence was 15.2 months, 91% of relapses occurred within 3 years. Frequent alarming symptoms were pain, weight loss and loss of appetite. Fifty-four percent recurred at multiple sites, 36% at a single anatomic site, and 10% were solitary. Recurrence was a 99% fatal event with a median overall survival (OS) of only 4.6 months. Older age, ypN3 at surgery, poor performance status, weight loss, non-solitary recurrence, no postoperative CTx, and no palliative CTx, were associated with short OS. Three patients had initial surgery, but all progressed; one additional patient was cured by salvage surgery after palliative CTx. Sixty percent (53 patients) were treated with CTx yielding a median progression-free survival and OS of 4.0 and 5.8 months, respectively; the overall response rate was 35%. Pleuroperitoneal metastases predicted poor prognosis. Non-platinum-based, first-line palliative CTx was used in 38%, mostly in patients with short treatment-free interval.

**Interpretation:**

In this national cohort, recurrence was a 99% fatal event and only 60% of patients received palliative CTx. Efficacy of palliative CTx at relapse after multi-modal treatment is poor and needs further investigations.

## Characteristics, treatment, and outcome of recurrent gastro-oesophageal adenocarcinoma after perioperative chemotherapy and radical resection

**Figure:**
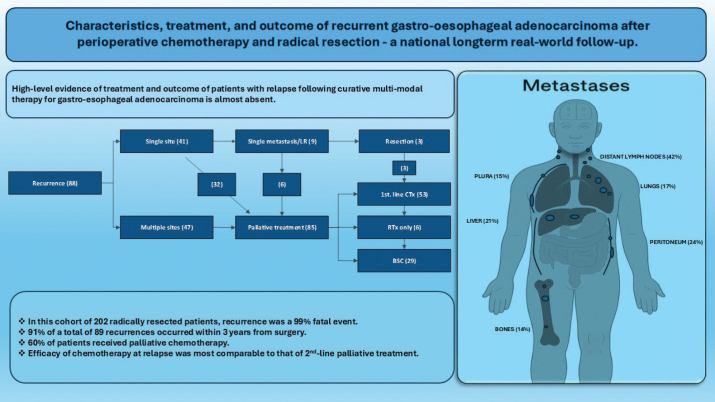

## Introduction

Gastro-oesophageal cancers (including oesophageal, gastro-oesophageal junctional, and gastric cancers) are associated with a high mortality, and gastric cancer is the fourth and oesophageal cancer the sixth most common causes of death from cancer globally [1, 2]. In contrast to declining incidence rates of gastric cancers, the incidence of adenocarcinomas at the gastro-oesophageal junction (GEJ) is increasing due to an association with obesity [2]. In Western countries without screening, only 40% of patients are diagnosed at a resectable stage [3, 4]. However, even with radical surgery, approximately 40% of patients will eventually experience recurrence [5].

Perioperative chemotherapy has been standard of care for resectable gastro-oesophageal adenocarcinoma since the publication of the MAGIC trial in 2006 [6]. However, pivotal studies have only sparsely described the fate of patients experiencing relapse [6, 7] despite that prior treatment may change the clinical presentation and biology of recurrent disease [8]. In patients relapsing after chemotherapy and surgery, drug resistance, previously experienced toxicity and treatment sequelae may play a major role for efficacy and feasibility of palliative treatment [9, 10]. In addition, in gastro-oesophageal cancer, most randomised studies of first-line palliative chemotherapy include almost exclusively treatment-naïve patients [11, 12], and therefore high-level evidence for benefit of investigated regimens in patients with relapse following multi-modality treatment is absent [13].

Up to half of patients diagnosed with recurrence never receive specific treatment [5] and may not even be referred to hospital units [14]. Consequently, studies on cohorts of relapsing patients that are hospital-based are biased towards better outcome. In this audit, that included a nationwide cohort, we wanted to investigate the characteristics, treatment and outcome of unselected patients experiencing recurrence after perioperative chemotherapy and radical resection for gastro-oesophageal adenocarcinoma. Such information may have an impact on, for example, the design of follow-up programmes, treatment of recurrent disease, and prognostic assessments.

## Materials and methods

### Patients

Through the Danish Esophago-Gastric Database (DEGD), and supplemented by local registers at all four hospitals treating gastro-oesophageal cancer in Denmark, we identified a consecutive, nationwide cohort of 202 patients that were radically resected (R0) after initiating perioperative chemotherapy with cisplatin or oxaliplatin, epirubicin, and 5-fluorouracil (FU)/FU-analogues for resectable gastro-oesophageal adenocarcinoma from the 1^st^ of May 2008 to the 29^th^ of June 2010. R0-resected patients constituted 71% of patients initiating perioperative chemotherapy [15].

In this cohort, with a minimum follow-up of surviving patients of 12 years, patients with a diagnosis of recurrence were identified from electronic health records (EHRs). We followed the European Society of Medical Oncology (ESMO) guidelines for reporting oncology real-world evidence (GROW) guidelines [16].

According to Danish national guidelines, patients attended regular clinical follow-up at the specialised departments, typically every 3^rd^ month the first year, then every 6^th^ month the next 1–2 years. There were no specific recommendations on follow-up after 3 years. Clinical assessment was combined with imaging, endoscopy, or blood tests, in general only if recurrence was clinically suspected.

### Clinico-pathological data

From EHRs we extracted data on site of primary tumor, post-neoadjuvant pathological (yp) stage, first alarming symptoms, diagnostic work-up, age, weight loss, and Eastern Cooperative Oncology Group (ECOG) performance status (PS) at recurrence, recurrence sites and subsequent treatment. For patients receiving palliative chemotherapy data were collected on regimen and date of progression. In patients with evaluable and measurable disease, response was retrospectively assessed from radiological reports and EHRs according to response evaluation criteria in solid tumours version 1.1 (RECIST) [17]. Adherence to postoperative chemotherapy and prior cumulated dose of perioperative platinum was also registered, and relative dose (RD) of perioperative platinum administered compared to standard full dosing was calculated for each patient. Finally, the time from end of perioperative chemotherapy to recurrence and start on palliative chemotherapy was assessed.

### Endpoints

The primary study endpoints were overall survival (OS) from date of recurrence and progression-free survival (PFS) from start of palliative chemotherapy. Other endpoints were OS from start of palliative chemotherapy, and overall response rate (ORR) of treatment.

### Statistical methods

Univariable Cox regression analyses were conducted with OS or PFS as the dependent variable and each of the baseline characteristics as the independent variable. A multivariable Cox regression analysis of OS was conducted and included factors significantly associated with OS in the univariable Cox regression analysis, but discarded ECOG PS and weight loss due to large numbers missing. Wald test *p*-values were reported to assess the statistical significance of the independent variables. Kaplan–Meier plots were constructed for selected variables, and differences among curves analysed by a log-rank test.

## Results

**Figure 1:**
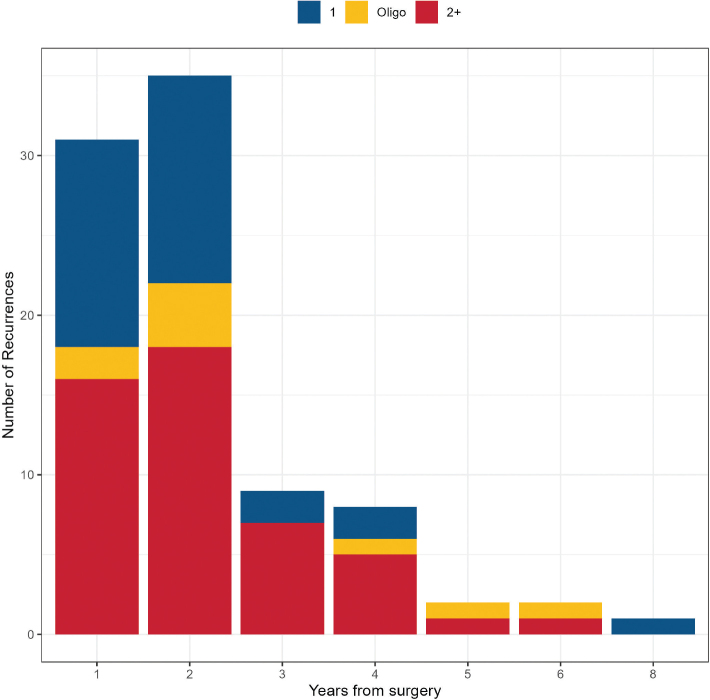
Distribution of recurrences per year after radical surgery and perioperative chemotherapy for gastro-oesophageal adenocarcinoma. Red: recurrence at multiple anatomical sites; Yellow: solitary metastasis/local recurrence only; Blue: recurrence at one anatomical site excluding solitary metastasis/local recurrence only. One patient diagnosed with recurrence at autopsy excluded.

In total, 89 (44.1%) patients were identified with recurrence during follow-up after radical resection and preoperative chemotherapy. Median time to recurrence from radical surgery was 15.1 months (range, 2.8 months to 7.2 years). Illustrated in Figure 1, 34.8% of recurrences occurred within 1 year from surgery, 74.2% within 2 years, and 91.0% within 3 years. Recurrence was a 99% fatal event and, thus, follow-up was virtually complete. Excluding the single surviving patient, the time from recurrence to death was a median of 4.6 months.

**Figure 2:**
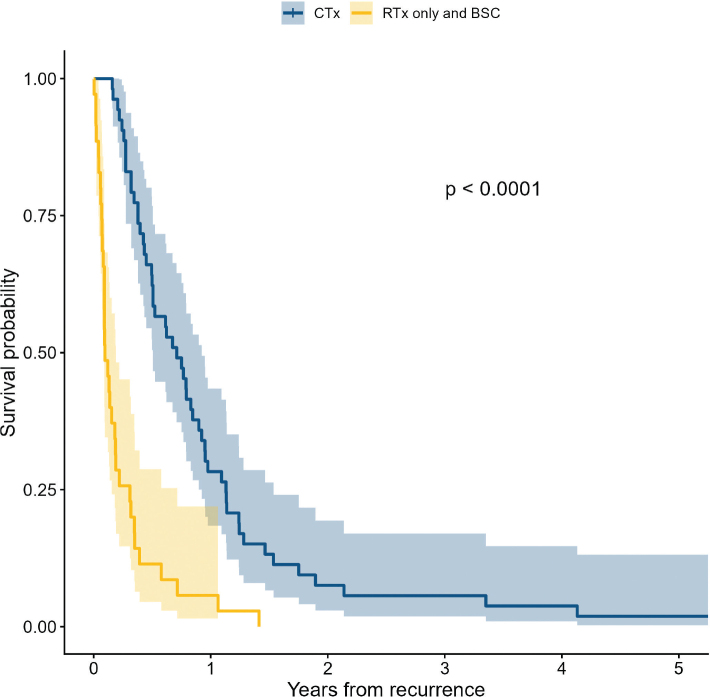
Overall survival of patients with recurrent gastro-oesophageal adenocarcinoma according to treatment. Blue: patients receiving palliative chemotherapy (CTx) (*N* = 53); Yellow: patients receiving best supportive care (BSC) or palliative radiotherapy (RTx) only (*N* = 35). One patient diagnosed with recurrence at autopsy excluded.

The patients’ characteristics and results of OS analysis are shown in Table 1. Median age at recurrence was 65 years (range, 35–80 years), 79.5% were male, and 85.2% had primary tumours in oesophagus or GEJ. Almost ¼ had not received postoperative chemotherapy, and the median RD of platinum given perioperatively was 80.5% (mean 75.4%; range, 16.0–100%). In a monovariable analysis, factors significantly associated with short OS from date of recurrence were increasing age, ypN3-stage at surgery, ECOG PS 2 or 3, and weight loss >10%, while administration of postoperative chemotherapy, palliative chemotherapy and solitary recurrence were associated with better OS after recurrence. A Kaplan–Meier plot of OS for patients receiving best supportive care or palliative chemotherapy is shown in Figure 2. Multivariable analysis showed independent poor prognostic value of ypN3-stage, while palliative chemotherapy and solitary recurrence were prognostically favourable.

**Table 1:** Results of univariable and multivariable analysis according to overall survival from date of recurrence in 88 patients with gastro-oesophageal adenocarcinoma.

Variable^1^	Stratum	Number	Univariable OS analysis			Multivariable OS analysis^4^		
			HR	95% CI	*P*	HR	95% CI	*P*
Sex	Male	70	Ref.	-	-			
	Female	18	1.12	(0.67–1.89)	0.67			
Age at recurrence [65 years]	Continuous	88	1.02	(1.00–1.04)	**0.04**	1.01	(0.99–1.03)	0.29
Primary site	Oesophagus/GEJ	75	Ref.	-	-			
	Gastric	13	1.46	(0.80–2.66)	0.21			
ypT-stage at surgery	0/1	1/5	Ref	-	-			
	2	42	0.89	(0.38–2.11)	0.80			
	3	32	0.76	(0.32–1.84)	0.55			
	4	8	0.83	(0.29–2.41)	0.74			
ypN-stage at surgery	0	23	Ref.	-	-	Ref.	-	-
	1	45	0.96	(0.57–1.61)	0.88	1.55	(0.86–2.78)	0.14
	2	13	0.88	(0.44–1.74)	0.71	1.54	(0.73–3.27)	0.26
	3	7	2.55	(1.07–6.05)	**0.03**	3.99	(1.59–10.05)	**0.003**
Start postoperative CTx	Yes	68	Ref.	-	-	Ref.	-	-
	No	20	2.42	(1.43–4.10)	**0.001**	2.28	(0.83–6.25)	0.11
RD perioperative platinum [80.5%]	Continuous	88	0.99	(0.98–1.00)	0.057	1.01	(1.00–1.03)	0.13
Time from surgery to relapse [14.9 months]	Continuous	88	0.99	(0.97–1.00)	0.11			
Recurrence metastasis pattern	Single anatomic site^2^	31	Ref.	-	-	Ref.	-	**-**
	Solitary/local recurrence only	9	0.34	(0.16–0.74)	**0.007**	0.34	(0.15–0.76)	**0.009**
	Multiple sites	48	0.74	(0.47–1.16)	0.19	0.71	(0.43–1.17)	0.18
ECOG PS	0	13	Ref.	-	-			
	1	24	1.58	(0.78–3.18)	0.20			
	2	11	2.50	(1.09–5.77)	**0.03**			
	3	9	7.09	(2.79–18.0)	**< 0.001**			
	Missing	31	-	-	-			
>10% weight loss	No	39	Ref.	-	-			
	Yes	15	2.15	(1.14–4.05)	**0.02**			
	Missing	34	-	-	-			
Alarming symptoms	None	10	Ref.	-	-			
	Any	77	1.08	(0.56–2.10)	0.82			
	Missing	1	-	-	-			
Palliative treatment	CTx	53	Ref.	-	-	Ref.	-	-
	BSC^3^	35	4.23	(2.66–6.72)	**< 0.001**	4.50	(2.62–7.75)	**< 0.001**

BSC: best supportive care; CI: confidence interval; CTx: chemotherapy; ECOG PS: Eastern Cooperative Oncology Group performance status; GEJ: gastro-oesophageal junctional; HR: hazard ratio; OS: overall survival; RD: relative dose of intended dose; Ref.: reference value (=1.0); RTx: radiotherapy; yp: post-neoadjuvant pathological.

One patient diagnosed with recurrence at autopsy excluded.

Significant P-values in bold.

1 Median value in brackets.

2 Excluding solitary/local recurrence only.

3 Including palliative RTx without CTx.

4 Variables significant or marginally significant in univariable analysis were included in multivariable analysis, except for ECOG PS and weight loss that were not included due to large number of missing values.

Details of first alarming symptoms at recurrence and diagnostic workup are provided in Supplementary Table S1. Twelve patients (13.5%) had recurrence detected incidentally. The first alarming symptom in the remainder patients was pain in almost half and weight loss or loss of appetite in 40%. More disease-specific alarming symptoms were less frequent; dysphagia and vomiting were seen in 13% of symptomatic patients, whereas anaemia or bleeding, hoarseness, ascites, and icterus were rarely reported. Cerebral symptoms were present in 6.5%. The workup at suspicion of recurrence included in most cases CT scans (74.7%), combined with positron emission tomography (PET) scans in 27.6%, while 16.1% had a gastroscopy. Surgery led to diagnosis in six cases. Two thirds of recurrences were histologically confirmed, and all were adenocarcinomas. A total of 30.8% of 39 cases investigated were human epidermal growth factor 2 (HER2)-positive.

Most patients (53.9%) had recurrence at multiple sites, while the remainder had recurrence at only a single anatomic site. Of the latter, nine patients (10.1% of all) had a solitary metastasis or solitary local recurrence. Shown in Table 2 in descending order of frequency, sites of recurrence were distant lymph nodes, peritoneum, liver, lungs, pleura, and bones. Recurrence at the anastomosis and brain metastases were both seen in 7.9% of cases.

**Table 2:** Sites of recurrence in 84 patients with relapse of gastro-oesophageal adenocarcinoma.

Site	Number (frequency)
Distant lymph node	37 (41.6%)
Peritoneum	21 (23.6%)
Liver	19 (21.3%)
Lung	15 (16.9%)
Pleura	13 (14.6%)
Bone	12 (13.5%)
Regional lymph node/anastomosis	11 (12.4%)
Brain	7 (7.9%)
Muscle/skin/soft tissue	6 (6.7%)
Cicatrices/port hole	3 (3.4%)
Spleen	2 (2.2%)
Adrenal gland	2 (2.2%)
Pancreas	2 (2.2%)
Colon	2 (2.2%)
Other sites^1^	3 (3.4%)

Site not reported in five patients.

1 Other sites include vagina, oesophagus, and bladder.

**Figure 3:**
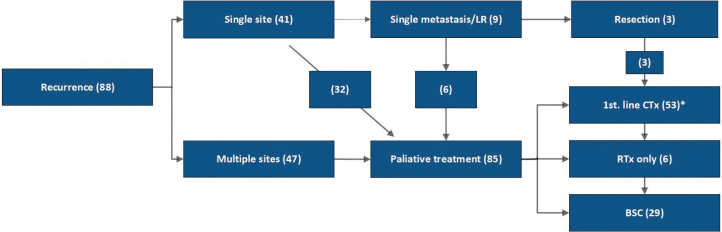
Distribution of treatment according to recurrence pattern in patients relapsing after surgery and perioperative chemotherapy for gastro-oesophageal adenocarcinoma. Number of patients in parentheses. One patient diagnosed at autopsy was excluded. *A total of 12 patients treated with CTx also received palliative RTx. Abbreviations: BSC, best supportive care; CTx, chemotherapy; LR, loco-regional recurrence only; RTx, radiotherapy.

Time from date of recurrence to start on antineoplastic palliative treatment was a median of 35 days (range, 0–143 days). The distribution of treatments according to recurrence pattern is shown in Figure 3. One third of patients with recurrence did not receive specific oncological treatment (median OS approx. 1 month), while six (6.7%) patients received only palliative radiotherapy (median OS 3.0 months). Twelve patients were treated by radiotherapy in addition to palliative chemotherapy. Primary indications for radiotherapy were pain (six cases), and spinal cord compression and central nervous system (CNS) metastasis in five cases each. Only three patients underwent initial, potentially curative surgical treatment, in one case preceded by neoadjuvant chemotherapy. All experienced relapse within 13.7 months from treatment of the recurrence. They proceed to palliative chemotherapy and were included in the cohort below from the date of 2^nd^ relapse. One additional patient with a solitary distant lymph node metastasis obtained a radiologically complete response to chemotherapy and received curative salvage surgery at progression of the lymph node metastasis.

A total of 53 patients (59.6%) received palliative chemotherapy. Shown in Supplementary Table S2, multiple regimens were used in 1^st^ line: Two-thirds of patients received platinum-based combination chemotherapy, including treatment in a randomised phase II study [18]. In 10 patients with HER2 overexpression, platinum-based chemotherapy was combined with trastuzumab. One fourth of patients received irinotecan, either single or combined with FU/FU-analogues, or with cetuximab in a phase II clinical study [19]. Seven patients were treated with paclitaxel, in five of these as a single drug. Only eight patients proceeded to 2^nd^ line chemotherapy, three received 3^rd^ line, and one patient received 4^th^ line chemotherapy. Patients treated with non-platinum-based chemotherapy had shorter interval from end of perioperative chemotherapy to start on palliative chemotherapy (median 7.9 vs. 17.7 months, *P* < 0.001), while a trend towards non-platinum-based 1^st^ line palliative chemotherapy versus platinum-based being more frequently used in patients having higher RD of perioperative platinum was observed (median RD 98.3% vs. 82.2%, *P* = 0.09).

**Figure 4:**
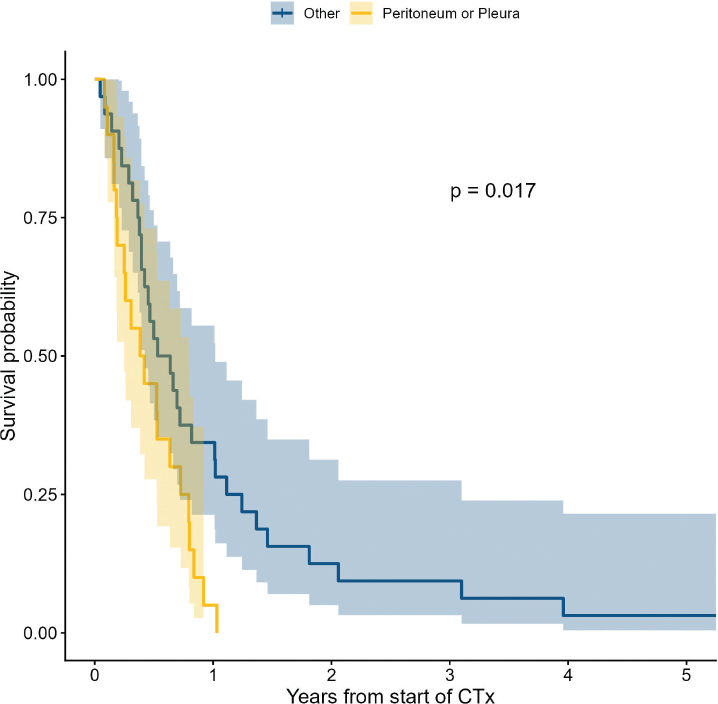
Overall survival of patients receiving palliative chemotherapy for recurrent gastro-oesophageal adenocarcinoma according to pleuroperitoneal involvement. Blue: patients without pleuroperitoneal metastasis (*N* = 32); Yellow: patients with pleuroperitoneal metastasis (*N* = 20). One patient treated with preoperative chemotherapy (CTx) with curative intent for recurrence excluded.

For patients receiving palliative chemotherapy, median PFS from the treatment start was 4.0 months (interquartile range (IQR) 2.0–7.5 months) and median OS was 6.2 months (IQR 3.4–10.5 months). Results of outcome analysis are shown in Supplementary table S3. In this analysis, several clinico-pathological and treatment characteristics were included but none significantly predicted PFS. An unfavourable OS was associated with recurrence situated in pleura or peritoneum (*P* = 0.02) (Figure 4), while prior weight loss was marginally significant (*P* = 0.09). A total of 26 patients had disease that was evaluable for response assessment at baseline. During 1^st^ line chemotherapy nine achieved an objective response, 10 had stable disease (SD), and three were not evaluated, accounting for an ORR of 34.6% (95% confidence interval [CI] 17.2–55.7%). Responders to palliative chemotherapy tended to have received lower prior perioperative platinum doses than non-responders (median RD 75.6% vs. 88.2%, *P* = 0.098).

## Discussion

This national audit of a Western population shows that recurrence after perioperative chemotherapy and radical resection for gastro-oesophageal adenocarcinoma is a fatal event with a median OS of only 4.6 months. At recurrence, approximately one third of patients received no specific treatment. This fraction was similar in a multicentre study of 4,682 patients with recurrent oesophageal cancer [20] but higher in a Dutch report of 858 patients relapsing after curatively intended treatment for cancer in oesophagus and GEJ, where more than half of patients received best supportive care (BSC) only [5]. These patients have a very short survival with high impact on the OS estimate. Patients treated with palliative chemotherapy had better OS from date of recurrence, although some immortal time bias should be considered. Patients with gastric adenocarcinomas constituted only 15%, reflecting the rarity of this diagnosis in the population studied compared to gastro-oesophageal junctional adenocarcinomas [21].

Prognostic and predictive factors known from studies of chemo-naïve patients may not be valid for pretreated populations [22]. Therefore, we assessed whether both the patients’ current status and prior characteristics had an impact on OS from diagnosis of recurrence. While high age, weight loss and poor PS are well recognised as clinically important prognostic factors with impact on patients’ ability to receive and benefit from palliative chemotherapy [23], we also found that ypN3-stage at surgery, non-solitary recurrence and non-adherence to postoperative chemotherapy were prognostically unfavourable, even after recurrence. Several explanations may account for this association. While ypN3 stage is a strong predictor for early recurrence [24], it seemingly also reflects an aggressive tumour phenotype after relapse. Non-solitary site recurrence may correlate with late diagnosis of relapse with a shorter lead time than recurrence at a solitary site, and perhaps also a more aggressive biology. Finally, failure to receive postoperative chemotherapy correlates with patients’ fragility, complications or resistance to prior treatment, and other poor prognostic factors, and likely also impact patients’ ability to receive and benefit from chemotherapy in the palliative setting.

Approximately 60% of patients in the current cohort received palliative chemotherapy. Outcome was poor with a median PFS of 4.0 months and a median OS of 5.9 months, shorter than reported in clinical trials of 1^st^ line palliative chemotherapy of approximately 6 and 11 months, respectively [25]. In this small cohort we were unable to identify factors predicting PFS, while according to OS, only pleuroperitoneal recurrence predicted poor outcome. Results suggest a limited benefit of palliative chemotherapy, and patients relapsing after perioperative chemotherapy probably should be regarded by oncologists as most similar to those receiving 2^nd^ line palliative treatment [26, 27]. Reflecting a significant knowledge gap, current ESMO guidelines provide no specific recommendations on choice of treatment regimen at recurrence after multimodal therapy [28].

The poor efficacy of chemotherapy has several possible causes. Persisting toxicities or experienced intolerance to perioperative chemotherapy may preclude many patients from receiving efficient doses of palliative chemotherapy at recurrence [29]. In addition, drug resistance may be acquired during perioperative treatment [30, 31]. In this study, these challenges were reflected by a trend towards a lower response rate of palliative chemotherapy in patients with high prior exposure to platinum as well as non-platinum-based 1^st^ line palliative chemotherapy was used more often in patients relapsing shortly after end of perioperative chemotherapy. Such issues may today be further accentuated by the later introduced fluorouracil/leucovorin/oxaliplatin/docetaxel (FLOT) regimen that in addition may confer resistance or intolerance to taxanes [31]. Hence future studies are warranted to guide clinicians in choosing the best palliative regimen at relapse, including new targeted treatment possibilities that have evolved recently and could improve outcome but were not available for the present cohort [13, 28].

In cohorts with follow-up without scheduled imaging, recurrence is symptomatic in 90% of patients [20]. We recorded the alarming symptoms leading to suspicion of recurrence and investigations performed at diagnosis of recurrence. Pain was the most common alarming symptom; however, constitutional symptoms were also frequent. Such symptoms are often associated with large tumour burden [32, 33], reflected by the finding of few patients with solitary recurrence and a large fraction of patients who were unable to receive palliative chemotherapy.

Although symptomatic patients have poor outcomes, the benefit of systematic follow-up of radically resected patients is still subject to debate and may depend on tumour and treatment characteristics [34, 35]. A large comparative observational study showed that radiological follow-up after surgery for oesophageal cancer had impact on OS only in patients with lower ypT-stages and in patients treated solely by surgery. However, the follow-up increased patients’ anxiety [5, 20]. Despite the fact that more patients can be offered active treatment [20], and the common assumption that early treatment initiation will result in OS benefit, there is no firm evidence, and prospective, randomised studies are needed.

In this audit, sites involved at recurrence were as expected with gastro-oesophageal adenocarcinomas [36], including cohorts of patients treated with FLOT [37]. Early sites of recurrence are partly dependent on primary tumour site as early recurrences from gastric cancer are most frequently localised to peritoneum, whereas gastro-oesophageal junctional or oesophageal adenocarcinomas often spread early to the liver [38]. In this cohort, a solitary metastasis or solitary locoregional recurrence was seen in only 10.1%. Locoregional recurrence was found in 12.4%, however, gastroscopy was not systematically used at workup. Recurrences are less disseminated in patients followed up systematically [20]. In patients with oesophageal and GEJ cancers treated with perioperative chemotherapy in the NeoAEGIS trial [39], 25% of 72 relapsing patients had locoregional recurrence, while in a Dutch population-based study, locoregional recurrence was observed in 18.8% [5].

Curative-intent treatment of local recurrence and oligometastatic relapse has primarily been studied in highly selected populations and results are divergent [40, 41]. In our national cohort, only three patients were offered curative surgery at recurrence, and all relapsed. We did not register non-surgical, potentially curative local treatment modalities such as high dose radiotherapy, chemoradiotherapy, or radio frequency ablation, however, no patients were cured by such treatment. Results are in accordance with a retrospective study from a single institution of 210 patients with gastro-oesophageal adenocarcinoma diagnosed from 2011 to 2016, where salvage therapy was attempted in 15 patients, but only four (1.9%) were successful [42]. Results of salvage therapy may be better in more modern cohorts. In a prospective database study of 246 patients diagnosed from 2014 to 2021, 27 patients received local therapy (irradiation or surgical intervention) for oligometastatic relapse, resulting in a prolonged OS in comparison to patients without local therapy (median OS 35.2 months vs. 7.8 months, *P* < 0.0001) [43].

This study has limitations mainly due to its retrospective nature, extracting data from EHRs. Furthermore, the small numbers in subgroups of patients and some missing clinical variables limits the statistics. The study includes patients with both oesophageal, GEJ and gastric tumours that may differ in biology and response to treatment [44, 45], and we had no information on molecular predictive or prognostic factors besides HER2 expression. We did not include patients treated with chemoradiotherapy [46] or upfront surgery with or without adjuvant treatment. The investigated perioperative treatment regimen is now largely obsolete [28] as well as new and more efficient palliative regimens have been introduced, warranting for future studies of relapsing patients treated by modern regimens. A recent report of 113 patients treated with perioperative FLOT showed a short median time to recurrence of 7 months among 28 patients experiencing recurrence, with most cases (61%) within the first year, suggesting a more aggressive course [37]. In another retrospective study of 196 patients treated with perioperative FLOT, half of recurrences was within 1 year, and the median OS of recurrent patients was 4.1 months from surgery, comparable to the outcome in our cohort despite more modern palliative treatment being available. In that cohort ypN3 at surgery was an unfavourable prognostic factor at relapse similar to our findings [24]. Despite different chemotherapy, we therefore believe that general findings and conclusions in this study are relevant for modern patients.

## Conclusion

This population-based audit of patients with recurrence after perioperative chemotherapy and radical resection of gastro-oesophageal adenocarcinoma that were followed-up with work-up, only when recurrence was clinically suspected, demonstrates the fatal consequence of relapse. Using this approach at follow-up, focus should be on improving patients’ quality of life rather than curation at relapse. Palliative chemotherapy had poor efficacy, most similar to that of 2nd line treatment and could only be offered to 60% of patients. Further studies are needed to better guide selection of palliative treatment in patients with relapse, who have previously been exposed to chemotherapy in the curative setting.

## Disclosure statements

ML received an unrestricted research grant from Scandion Oncology A/S, Copenhagen, Denmark, and is advisory board member at Alivia SE, Stockholm, Sweden. The remaining authors disclosed no conflicts of interest.

## Supplementary Material





## Data Availability

Anonymous data of the clinical database can be provided upon reasonable request to the corresponding author.
