# Supplementary material for: Characteristics, treatment, and outcome of recurrent gastro-oesophageal adenocarcinoma after perioperative chemotherapy and radical resection
Source: Acta Oncol. 2026 Jan 11;65:44264. doi: 10.2340/1651-226X.2026.44264 (PMC12801056; doi:10.2340/1651-226X.2026.44264)
Supplement: Supplementary file 2 [file AO-65-44264-s2.pdf]

Supplementary material has been published as submitted. It has not been copyedited, or typeset by Acta Oncologica

Supplementary Table S1a. First alarming symptom in 77 symptomatic patients prior to detection of recurrence.

Symptom	Number (frequency)
Pain	38 (49.4%)
Weight loss/loss of appetite	31 (40.3%)
Reduced performance/astenia	11 (14.3%)
Dysphagia/vomiting	10 (13.0%)
Dyspnoea	6 (7.8%)
Cerebral symptoms	5 (6.5%)
Dyspepsia	3 (3.9%)
Ascites	2 (2.6%)
Icterus	2 (2.6%)
Abnormal blood tests	2 (2.6%)
Bleeding/anaemia	2 (2.6%)
Ileus	2 (2.6%)
Other*	3 (3.9%)
Not specified	7 (9.1%)

Symptoms were not assessed in 3 patients.

*Other includes one each of cardiac arrythmia, hoarseness, and palpable tumour.

A total of 44 patients (57.1%) had one symptom, 20 (26.0%) two symptoms, and 13 (16.9%) three alarming symptoms. Twelve patients were diagnosed incidentally.

Supplementary Table S1b. Workup at recurrence in 87 patients.

Investigation	Number (frequency)
CT	65 (74.7%)
Added PET	24 (27.6%)
Gastroscopy	14 (16.1%)
Ultrasound	9 (10.3%)
Surgery	6 (6.9%)
Pleuracentesis or ascites puncture	6 (6.9%)
MRI	4 (4.6%)
CTC or MRIC	3 (3.4%)
X-ray	1 (1.1%)
Other*	2 (2.3%)

Not assessed in 2 patients.

*Other includes one diagnosed at autopsy and one not specified.

45 (51.7%) had one investigation, 34 (39.1%) two investigations, and 8 (9.2%) three investigations at workup. 58 (66.7%%) of recurrences were histologically or cytologically verified.

CTC, CT scan of cerebrum; CT, computer tomography; MRI, magnetic resonance imaging; MRIC, magnetic resonance imaging of cerebrum; PET, positron-emission tomography.

Supplementary Table S2. 1st line palliative chemotherapy regimens.

	**Number of patients**
**Platinum-based** (62.2%)*	
Oxaliplatin + capecitabine/5FU	11
Oxaliplatin + epirubicin + capecitabine	10
Carboplatin + docetaxel + capecitabine	7
Oxaliplatin + docetaxel + capecitabine/S1	4
Carboplatin + paclitaxel	1
**Irinotecan-based** (24.5%)	
Irinotecan	6
Irinotecan + cetuximab	5
Irinotecan + 5FU/capecitabine	2
**Taxane-based** (13.2%)	
Paclitaxel	5
Paclitaxel + ramucirumab	1
Paclitaxel + capecitabine	1

*Added trastuzumab in 10 cases.

FU, fluorouracil.

Supplementary table S3. Results of univariate analysis according to progression-free and overall survival from date of treatment start in 52 patients receiving 1st line palliative chemotherapy after perioperative chemotherapy and radical surgery for gastroesophageal adenocarcinoma.

Variable[1]	Stratum	Number	Overall survival			Progression-free survival		
			HR	95%CI	P-value	HR	95%CI	P-value
Gender	Male	42	Ref.	-	-	Ref.	-	-
	Female	10	0.86	(0.43-1.73)	0.68	0.95	(0.47-1.93)	0.89
Age [62 years]	Continuous	52	1.02	(0.99-1.04)	0.24	1.02	(0.99-1.05)	0.30
Primary site	Oesophagus/GEJ	45	Ref.	-	-	Ref.	-	-
	Gastric	7	1.23	(0.55-2.76)	0.62	1.03	(0.46-2.32)	0.94
Time from end of perioperative CTx to start on palliative CTx [14.2 months]	Continuous	52	1.00	(0.98-1.02)	0.88	0.99	(0.97-1.01)	0.49
RD of perioperative platinum [83.3%]	Continuous	52	0.99	(0.98- 1.01)	0.51	1.00	(0.98-1.01)	0.68
ECOG PS (12)	0	12	Ref.	-	-	Ref.	-	-
	1	23	1.56	(0.75-3.23	0.23	1.33	(0.65-2.75)	0.44
	2-3	5	1.57	(0.53-4.69)	0.42	1.05	(0.33-3.35)	0.93
Weight loss >10% (12)	Yes	10	Ref.	-	-	Ref.	-	-
	No	30	0.52	(0.24-1.12)	***0.09***	0.60	(0.29-1.25)	0.17
Site of recurrence	Liver vs other	12/40	0.74	(0.39-1.43)	0.37	0.67	(0.34-1.29)	0.23
	Distant lymph nodes vs other	25/27	0.76	(0.43-1.33)	0.34	0.92	(0.53-1.62)	0.78
	Pleuroperitoneum vs other	20/32	2.07	(1.13-3.82)	**0.02**	1.38	(0.78-2.45)	0.27
Number of sites of recurrence	1	21	Ref.	-	-	Ref.	-	-
	2	21	1.02	(0.54-1.91)	0.95	0.88	(0.47-1.64)	0.68
	3-4	10	1.19	(0.55-2.60)	0.66	1.20	(0.55-2.59)	0.65
Type of 1st line palliative CTx	Platinum-based	33	Ref.	-	-	Ref.	-	-
	Non-platinum-based	19	1.16	(0.65-2.08)	0.61	1.26	(0.70-2.25)	0.44

One patient treated with preoperative chemotherapy with curative intent for recurrence was excluded.

1) Median value in brackets, missing number in parentheses.

CI, confidence interval; CTx, chemotherapy; ECOG PS, Eastern Cooperative Oncology Group performance status; GEJ, gastroesophageal junctional; HR, hazard ratio; RD; relative dose of intended dose; Ref., reference value (=1.0).
